# A meta-analysis into the mediatory effects of family planning utilization on complications of pregnancy in women of reproductive age

**DOI:** 10.1371/journal.pone.0294475

**Published:** 2024-03-18

**Authors:** Shayesteh Jahanfar, Olivia Maurer, Amy Lapidow, Anjali Rajkumari Oberoi, Meredith Steinfeldt, Moazzam Ali

**Affiliations:** 1 Department of Public Health and Community Medicine, Director of Tufts University, Boston, MA, United States of America; 2 Affiliate of Cochrane US, Tufts University School of Medicine, Boston, MA, United States of America; 3 School of Medicine, Tufts University School of Medicine, Boston, MA, United States of America; 4 Department of Public Health and Community Medicine, Tufts University School of Medicine, Boston, MA, United States of America; 5 WHO Department of Sexual and Reproductive Health and Research, World Health Organization, Geneva, Switzerland; Innovative Aid, CANADA

## Abstract

**Background:**

Despite conflicting findings in the current literature regarding the correlation between contraceptives and maternal health consequences, statistical analyses indicate that family planning may decrease the occurrence of such outcomes. Consequently, it is crucial to assess the capability of family planning to mitigate adverse maternal health outcomes.

**Objectives:**

This review investigates the effects of modern contraceptive use on maternal health.

**Search methods:**

This systematic review is registered on Prospero (CRD42022332783). We searched numerous databases with an upper date limit of February 2022 and no geographical boundaries.

**Selection criteria:**

We included observational studies, including cross-sectional, cohort, case-control studies, and non-RCT with a comparison group. We excluded systematic reviews, scoping reviews, narrative reviews, and meta-analyses from the body of this review.

**Main results:**

The review included nineteen studies, with five studies reporting a reduction in maternal mortality linked to increased access to family planning resources and contraceptive use. Another three studies examined the impact of contraception on the risk of preeclampsia and our analysis found that preeclampsia risk was lower by approximately 6% among contraceptive users (95% CI 0.82–1.13) compared to non-users. Two studies assessed the effect of hormonal contraceptives on postpartum glucose tolerance and found that low-androgen contraception was associated with a reduced risk of gestational diabetes (OR 0.84, 95% CI 0.58–1.22), while DMPA injection was possibly linked to a higher risk of falling glucose status postpartum (OR 1.42, 95% CI 0.85–2.36). Two studies evaluated high-risk pregnancies and births in contraceptive users versus non-users, with the risk ratio being 30% lower among contraceptive users of any form (95% CI 0.61, 0.80). None of these results were statistically significant except the latter. In terms of adverse maternal health outcomes, certain contraceptives were found to be associated with ectopic pregnancy and pregnancy-related venous thromboembolism through additional analysis.

## Introduction

Globally, 966 million women of reproductive age are using some method of contraception. However, an estimated 164 million women who want to avoid pregnancy, meaning they do not want to become pregnant for at least two years or stop childbearing altogether, are not using any contraceptive method, meaning that they are considered to have an unmet need for family planning [1]. There are many possible explanations for this widespread unmet need for family planning, including awareness and access issues, issues related to method use, opposition by the woman or a family member, or sexual activity patterns [2] Click or tap here to enter text.

Unsafe abortions account for 13% of maternal deaths and 94% of maternal deaths occur among women living in low-resource settings [3]. Results from modeling studies, such as those from Stover et. al [4] and Ahmed et al. [5], have predicted substantial drops in maternal mortality rates internationally upon increasing contraceptive use. This research suggests that expanding family planning resources is a powerful tool that can move the international community toward achieving the sustainable development goal of reducing the global maternal mortality ratio to less than 70 per 100,000 live births [6, 7] Click or tap here to enter text. Cleland et al [8] noted that a further 30% of maternal deaths could be avoided by fulfilment of unmet need for contraception. Our review aims to complement these previous studies by providing data from populations that have been affected since the publication of this work in 2012 so that the results can be compared.

We found that several existing reviews focused on the determinants of contraceptive use in LMIC [9–11]. These reviews focused on a specific subgroup of contraceptive users, such as those with HIV or those with diabetes [12–14]. Our review will contribute information that is more generalizable.

### Objectives

Our review will focus on both beneficial and adverse reproductive health outcomes. Ectopic pregnancy, for example, is the implantation of an embryo outside the uterus and is a relatively rare, albeit life-threatening gynecological emergency. Approximately 1–2% of all naturally conceived pregnancies fall into this category [15]. Specifically, research suggests that the use of the IUD may increase a woman’s risk of conceiving an ectopic pregnancy (OR 4.38, CI [1.78–10.81, P = 0.001]) [16]. This is not to suggest that the IUD is not a viable method of contraception for many users. However, it is important to report the protective effects of all types of contraceptives as well as risks associated with their use so that women and their providers can make well-informed decisions surrounding reproductive health.

Additional maternal morbidities that may be affected by contraceptive use are hypertensive disorders of pregnancy. The relationship between contraceptive use and hypertensive disorders of pregnancy is not entirely understood. One study found that the recent use of oral contraceptives was associated with a reduced risk of developing gestational hypertension (OR 0.7 (95% CI, 0.4–1.0)). In contrast, there was a suggestion that recent use was associated with an increased risk of developing preeclampsia, but only among women who had used oral contraceptives for 8 years or more (OR 1.3, 95% CI, 0.8–2.4) [17].

## Material and methods

### Protocol and registration

This systematic review is registered on Prospero (CRD42022332783). It is focused on quantitative evidence on human subjects with reportable outcomes and measurement of risk of reproductive health outcomes among women of reproductive age (15–49 years of age).

### Eligibility criteria

We included all family planning methods that the WHO defines as effective and acceptable, including 1) short-acting hormonal contraception (e.g., birth control pills, patches, vaginal rings), 2) Long-term contraception (e.g., hormonal, or non-hormonal intrauterine devices, implants, injections, 3) one-time barrier contraception (e.g., condoms, sponges, diaphragms, cervical caps, spermicide), 4) permanent contraception (Tubal ligation and vasectomy) 5) emergency contraception (morning after pill, or intrauterine devices).

### Information sources

We searched the CINAHL (1981 - https://tischlibrary.tufts.edu/what-we-have/databases/cinahl), OVID Medline (1946 - https://tischlibrary.tufts.edu/what-we-have/databases/cinahl), EMBASE (1947 - https://www.elsevier.com/products/embase/content), Psycho INFO (1800s - https://www.apa.org/support/psycinfo), Maternity & Infant Care (1857 - https://www.midirs.org/resources/maternity-and-infant-care-mic-database/), LILACS (1982 - https://lilacs.bvsalud.org/en/#about), clinical trial.gov (2008 - https://www.ncbi.nlm.nih.gov/pmc/articles/PMC8591666/), web of science (1900 - https://clarivate.com/products/scientific-and-academic-research/research-discovery-and-workflow-solutions/webofscience-platform/), SCOPUS (2004 - https://www-elsevier-com.ezproxy.library.tufts.edu/products/scopus/content?dgcid=RN_AGCM_Sourced_300005030), and CENTRAL Database (1996 - https://www.cochranelibrary.com/central/about-central) until February 2022, with no geographical boundaries. We also included WHO local databases as follows: Africa (AIM), Latin America and the Caribbean (LILACS), A network of Health Science Libraries across Asia (HELLIR), Virtual Health Sciences Library, IBECS (ibecs.isciii.es)

SciELO (Scientific Electronic Library Online; www.scielo.br), LILACS (Latin American and Caribbean Health Sciences Literature; lilacs.bvsalud.org/en), PAHO (Pan American Health Library; www1.paho.org/English/DD/IKM/LI/library.htm), WHOLIS (WHO Library; dosei.who.int), WPRO (Western Pacific Region Index Medicus; www.wprim.org), Index Medicus for the South‐East Asia Region (IMSEAR; imsear.hellis.org), IndMED (Indian medical journals; indmed.nic.in; 1985 onwards), Native Health Research Database (hscssl.unm.edu/nhd/). (See S1 Appendix)

**Fig 1:**
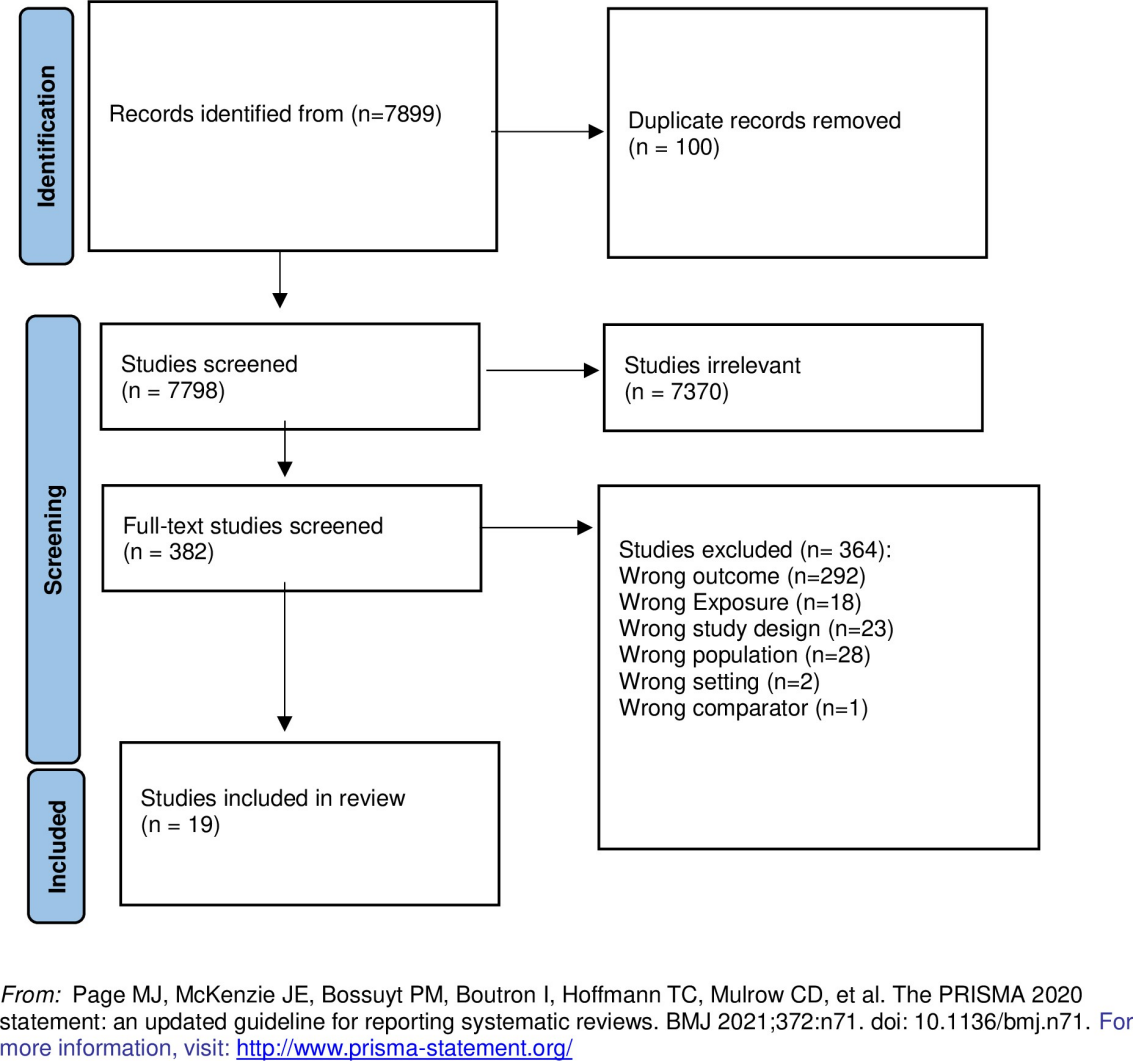
PRISMA flow diagram.

Moreover, we searched websites of societies, universities, and institutions devoted to contraception use and family planning, which are included in the appendix. We checked the references of the reviews and references of the original RCTs to ensure no original study was missed. Our Health Librarian designed our search strategy at Tufts, Ms. Amy Lapidow. This search strategy was approved by Dr. Ali Moazzam from WHO. The search strategy can be found attached (See S2 Appendix). A PRISMA flow diagram is used to show the selection of the studies. The reasons for excluding the full-text papers were documented in the PRISMA flow diagram (Fig 1) and reported in the findings section throughout the screening process.

### Search

First, we conducted a Medline search. The medical subject heading (MeSH) search terms and keywords, used either independently or in combinations, are included in the appendix.

Second, the Medline search strategy was adjusted to the dictionary of the other databases (CINAHL, OVID Medline, EMBASE, Psycho INFO, POLLINE, Web of Science, and CENTRAL Database, Science Citation Index Expanded (SCIEXPANDED), and WHOLIS) as applicable. We also searched the references of the full-text papers relevant to the review.

We used OpenGrey (www.opengrey.eu), Google, Google Scholar to search for relevant grey literature.

### Study selection

The primary exposure was contraceptive use and the comparison was considered no contraception use only. The outcomes of interest were maternal morbidity and maternal mortality. We sought to include studies with any of the following study designs: observational studies including cross-sectional, cohort, case-control studies, and any non-RCT with a comparison group.

### Data collection process

Our team screened the papers in pairs obtaining a Kappa score of more than 7. Two authors (OM, AO) independently screened the abstracts (stage 1) and the full papers (stage 2) using Covidence and resolved any conflicts by discussion. We then divided the full papers that had passed the first screening stage between the two reviewers to extract data. Data collection was handled outside of Covidence and using data extraction forms which were safely kept on Box (S3 Appendix). Summary tables were then created manually and data were entered into Revman for analysis.

### Data items

As for the population, we included women of reproductive age (15–49 years of age). We included the following family planning methods: 1) short-acting hormonal contraception (e.g., birth control pills, patches, vaginal rings), 2) Long-term contraception (e.g., hormonal, or non-hormonal intrauterine devices, implants, injections, 3) one-time barrier contraception (e.g., condoms, sponges, diaphragms, cervical caps, spermicide), 4) permanent contraception (Tubal ligation and vasectomy) 5) emergency contraception (morning after pill, or intrauterine devices). If the studies were observational, contraceptive use of all types was considered the primary exposure The comparison was considered no contraception use only. The outcomes of interest were maternal morbidity and maternal mortality. During our screening stage, we sought to include studies with any of the following study designs: observational studies including cross-sectional, cohort, case-control studies, and any non-RCT with a comparison group.

### Risk of bias in individual studies and across studies

We assessed the risk of bias (high, moderate, or low) in each included study following Down and Black criteria. For each study, we examined reporting, external validity, internal validity, and other areas for potential bias. Two authors independently assessed the risk of bias in each included study. This information was entered into Revman to create two risk of bias figures: risk of bias assessment for each study and bias assessment for the entire review.

### Summary measures, synthesis of results, and additional analyses

The analysis of this review was limited to the analytic method used in the study report (e.g., intent to treat, per protocol, or a modification of either type). Studies were combined for meta-analysis only when identical family planning devices/tools/drugs, dosages, and regimens were compared. We calculated odds ratios (OR) or mean difference (MD) with a 95% confidence interval (95%CI) for each dichotomous or continuous outcome, respectively. Subgroup analysis was conducted using different types of contraception, dose, and route of administration when possible. We conducted meta-analysis if we had two data points or more for each comparison and outcome. We assessed the homogeneity of studies combined in a meta‐analysis using both fixed‐effect and random‐effects models. Any score of I^2^ above 50% was investigated for the clinical and methodological diversity of the studies.

## Results and discussion

### Study selection and characteristics

In total, 19 studies were included. S4 Appendix shows some characteristics of these studies as follows: countries, year of publication, number of facilities, type of health facility, level of health facility, sample size, study design, population, type of contraception studied, the outcome of interest extracted, and quality of study based on proper study design. Most studies were from 2000 onward (n = 15). We included eight case-control studies, two prospective cohort studies, seven retrospective cohort studies, and two cross-sectional studies.

We then categorized the studies into the following outcome categories: Glucose Tolerance, Preeclampsia, High-Risk Pregnancy and Birth, Maternal Mortality, Pregnancy-Related Venous Thromboembolism, and Ectopic Pregnancy.

### Risk of bias within and across studies

Table 1 shows the quality assessment of observational studies using the Downs and Black Scoring [18]. We considered the overall quality of evidence to be moderate for our review. The mean methodological quality score was 15.4+/2.6 out of 28 on the Downs and Black checklist (median = 16, Min = 9, max = 19).

**Table 1:** Assessments based on the downs and black scoring system.

Author, year	Reporting	External validity	Bias	Confounding	Total score
Abdalhabib 2021	8	2	4	2	16
Bahamondes 2014	8	2	4	2	16
Bastani 2007	6	2	3	2	13
Bourke 2015	7	2	4	2	15
Chikandiwa 2018	7	3	4	3	17
Chowdhury 2007	8	3	5	3	19
Godefay 2015	6	3	4	3	16
Gupta 2019	7	0	3	1	11
Hedderson 2007	9	3	4	3	19
Lech 2005 [37]	7	0	3	2	12
Li 2015	7	2	3	3	15
Nelson 2008	9	0	4	3	16
No author listed 1998	9	0	3	2	14
Parker 2016	8	3	4	2	17
Petersen 2014 [38]	8	3	4	2	17
Ronsmans 1997	8	2	5	3	18
Skouby 1982	5	0	4	0	9
Shurie 2018	7	3	4	2	16
Thadhani 1999	7	3	4	3	17

### Results of individual studies

#### Maternal mortality

Five studies [19–23] analyzed the effect of contraceptive use on maternal mortality rates in each population. We were not able to pool the results due to the diversity of study design, and lack of comparison. The research team headed by Bahamondes [19] found that the provision of long-acting reversible contraceptive methods and DMPA injections prevented 37–60 maternal deaths and between 1056 and 1412 unsafe abortions averted, using disability-adjusted life years lost for these calculations.

Chowdhury and colleagues used data from the Matlab (Bangladesh) Demographic Surveillance System on 165,894 pregnancies over the period 1982–2005 to calculate measures of maternal mortality. Time trends in maternal mortality were estimated using logistic regression analyses [22]. Maternal mortality was reported to fall in areas who received contraceptives over 30 years [OR = 0.41, 95% CI 0.25–0.67]. Part of the decline was due to a fall in abortion-related deaths and better access to emergency obstetric care and trained birth attendants. Ronsmans and colleagues used the same set of data, finding that, after the onset of programming, direct obstetric mortality declined by 3% per year from 1976–1993, although regional discrepancies were apparent [RR 0.97/year 95% CI 0.95–0.99].

Gupta and colleagues conducted a study which showed that the introduction of the contraceptive implant on Karkar island led to a decrease in high-risk pregnancy characteristics such as short interpregnancy interval, postpartum infection, postpartum hemorrhage, and hospital readmission, which often resulted in maternal mortality [20]

Some of the limitations of this study are as follows:

1. Retrospective design: The study used birth records that were routinely collected, which may have limited the scope and accuracy of the data available. Retrospective studies are also subject to bias and confounding factors that may affect the accuracy of the findings.
2. Single-site study: The study was conducted on Karkar Island in Papua New Guinea, which may limit the generalizability of the findings to other settings.
3. Possible confounding factors: The study did not account for other factors that may have influenced the observed changes in birth outcomes, such as changes in healthcare access or other public health initiatives.

Finally, Godefay [21] aimed to identify risk factors for maternal mortality in rural Tigray, Ethiopia, using a case-control study design. The cases were 96 women who died during pregnancy, delivery, or within 42 days postpartum, and the controls were 192 women who survived childbirth. The data were collected through interviews with women’s relatives and healthcare providers and analyzed using logistic regression. Not ever use of contraception led to 158% higher odds of maternal death (95% CI 1.37–4.85). Some possible limitations of this study may include:

1. Generalizability: as the study was conducted in rural Tigray, it may not be generalizable to other regions or settings with different demographic, cultural, or healthcare system characteristics.
2. Sample size: the study may have had a limited sample size, which could affect the statistical power and precision of the results.
3. Potential confounding factors: the study may not have controlled for all potential confounding factors that could affect the relationship between the risk factors and maternal mortality.

### Maternal morbidity

#### Preeclampsia

**Fig 2:**
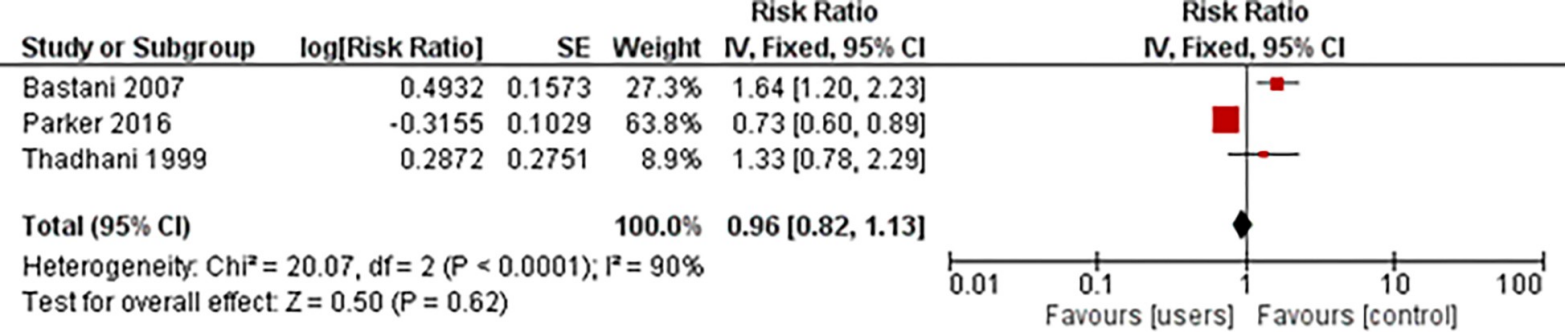
Risk of developing pre-eclampsia.

The three studies [24–26] found that using any form of contraception was associated with a 6% lower risk of developing preeclampsia compared to not using contraception, with a non-statistically significant 95% confidence interval ranging from 0.82 to 1.13. The analysis also exhibited a high level of heterogeneity. (Fig 2)

#### High-risk pregnancy and birth

**Fig 3:**
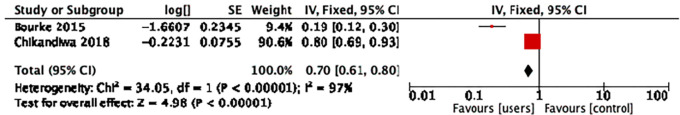
High-risk pregnancy and birth.

It was found in the review that two studies by Bourke [27] and Chikandiwa [28] examined the risk of high-risk pregnancies and births in women using contraceptives compared to those who did not. The results suggested that there was a 30% lower risk of high-risk pregnancies and births among women using any form of contraception (95% CI 0.61, 0.80). However, the studies showed a high degree of variation in the results with a heterogeneity of 97%. (Fig 3).

#### Pregnancy-related venous thromboembolism

**Fig 4:**
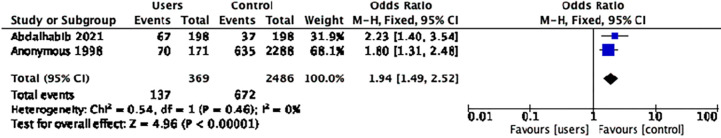
Pregnancy-related venous thromboembolism.

We found two studies that examined the impact of contraceptive use on the risk of venous thromboembolism (VTE) during pregnancy compared to non-users [29, 30]. The results indicated that there was a 94% higher risk of VTE among contraceptive users (95% CI 1.49, 2.52). Notably, there was no heterogeneity observed between the two studies. (Fig 4).

#### Ectopic pregnancy

**Fig 5:**
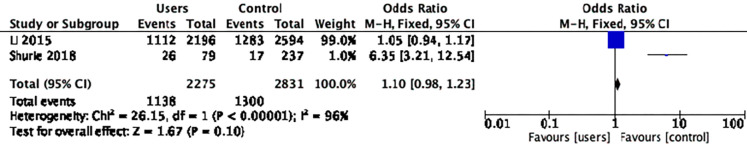
Emergency contraceptive use and ectopic pregnancy.

The effect measure reported for two studies [31, 32] investigating the relationship between emergency contraceptive use and ectopic pregnancy compared to non-users was not significant, with a OR of 0.93 (95% CI 0.42, 2.07) and a OR of 0.77 (95% CI 0.20, 2.93), respectively. This led to the summarized OR of 1.10 (0.98, 1.23) in the forest plot with high level of heterogeneity (96%). (Fig 5).

### Summary of evidence

#### Maternal mortality

Gupta and colleagues illustrated the protective effect of contraceptive implants on maternal morbidity and mortality [20]. Two other studies reported the associations between contraceptive use and maternal mortality, illustrating the protective effect of family planning against maternal mortality [19, 21]. Studies tended to attribute these findings to the increase in reproductive health literacy among contraceptive users and increased reproductive autonomy among users. To achieve a more comprehensive understanding of the impact of contraception on maternal health, alternative methods of assessment could be employed. These methods could include:

1. Mathematical modeling: This approach involves mathematical models that simulate the effects of contraception on maternal health outcomes.
2. Hospital-based or population-based studies: These studies involve collecting data from a large group of people in a specific geographic area or population.
3. Meta-analyses of existing data: Meta-analyses involve synthesizing data from multiple studies to provide a more robust analysis of the effects of contraception on maternal health outcomes. Meta-analyses can help to identify patterns and trends that may not be apparent in individual studies.

According to Chola et al., further modeling research suggests that scaling up family planning together with optimal maternal, newborn, and childcare is critical to the health of mothers and children [17]. The study found that "if contraceptive prevalence rate increased by just 0.68% annually, the number of pregnancies would reduce from 1.3 million in 2014 to one million in 2030.” Additionally, the researchers found that unintended pregnancies, abortions, and births would decrease by approximately 20%, preventing an estimated 600 maternal deaths [17]. This paper emphasizes the minimal investment that would be required of government agencies and policymakers to make improvements of great magnitude in reproductive health.

#### Importance of Covid-19 pandemic in this context

It is also important to note the effects of the COVID-19 pandemic on both contraceptive production and uptake. The health system was overloaded with COVID-19 cases and concerns, so many health facilities suffered in their ability to maintain adequate provision of reproductive health services. Additionally, many patients were afraid to go to health clinics for purposes other than acute illness. Researchers estimated that a 10% decline in the proportion of women receiving sexual and reproductive health services in low- and middle- income countries would result in an additional 49 million women having an unmet need for modern contraceptives and an additional 15 million unintended pregnancies during a period of one year [33]. This data suggests that part of our global effort to recover from the effects of COVID-19 must include a concerted effort in the reconstruction of reproductive health services so that we might avoid seeing an increase in maternal mortality rate.

#### Maternal morbidity

The studies conducted by Bourke and Chikandiwa observed a decrease in high-risk pregnancies and birth associated with prior contraceptive use [27, 28]. Three studies reported mixed results concerning the relationship between contraceptive use and preeclampsia. Thadhani et al. found that recent users of oral contraception ≥8 years were associated with 2.1 times the risk of developing preeclampsia compared to never or past users [26]. However, Parker et al. found that using an IUD before pregnancy was associated with a small reduction in the risk of preeclampsia [25]. Bastani et al. found that barrier and withdrawal methods of contraception were associated with a higher risk of preeclampsia, while women who had more frequent coitus before conception were at a lower risk of developing preeclampsia [24]. Thus, varying levels of risk of preeclampsia are associated with different types of contraception.

Overall, the findings of these studies suggest that healthcare providers need to consider the individual risk factors and timing of contraceptive use when working with women who have a history of gestational diabetes or are at risk of venous thromboembolism. Additionally, further research is needed to better understand the long-term effects of contraceptive use on maternal health outcomes, including glucose tolerance and venous thromboembolism, and to identify potential strategies for reducing these risks.

It is worth noting that despite the slight statistical effect of contraceptive use on postpartum glucose tolerance as reported by pooled analysis, there is little clinical evidence to support the need for intervention based on experimentally observed changes in blood glucose tolerance after initiating contraceptives [34–36]. Current evidence-based guidelines recommend monitoring relevant measures such as blood pressure, weight, body mass index, and blood glucose levels.

### Limitations

Although the review utilized a comprehensive search strategy, some relevant publications may have been excluded due to the specific inclusion criteria. Additionally, focusing only on studies that directly address the review’s specific aims and objectives may have resulted in the omission of other important topics related to maternal health and modern contraceptive use. The exclusion of modeling studies may have limited the review’s scope and potential insights into the impacts of contraceptive use on maternal health outcomes. Furthermore, the limited number of studies meeting the specific criteria may restrict the generalizability of the findings. Therefore, while the review provides valuable insights, it may not be comprehensive in terms of recommendations for improving women’s reproductive health outcomes through contraceptive use. Further research is necessary to expand the current knowledge in this field.

### Potential biases in the review process

There are several potential biases in the review process that need to be acknowledged. Firstly, as stated in the review, there was much variation in the incidence and prevalence of maternal morbidities and mortality between studies. This makes it difficult to draw accurate conclusions from the studies and limits the generalizability of the findings. Secondly, there was a variety of diagnostic criteria and outcome metrics used between studies. This can make it difficult to compare results between studies and to draw accurate conclusions. Thirdly, certain studies failed to report critical study, setting, and population characteristics, which may affect the comparability of their data. This makes it difficult to assess the quality and reliability of the studies. Finally, it is important to note that the studies included in the review were limited to those that were published up until the knowledge cutoff date of the review. As new studies are published, the conclusions and recommendations of the review may become outdated.

Our review contributes to the literature on reproductive health by including studies that take a broader approach and examine mothers at various points before, during, and after pregnancy. As such, healthcare providers and policymakers should prioritize increasing the availability and accessibility of family planning resources, with a focus on understanding the unique needs and challenges faced by women in different populations and settings.

## Conclusions

In conclusion, a comprehensive review of nineteen studies has highlighted the multifaceted impact of contraception on maternal health. Notably, increased access to family planning resources and contraceptive use was associated with a reduction in maternal mortality in five studies and a modest 6% decrease in preeclampsia risk. However, the type of contraception used appeared to influence postpartum glucose tolerance, with low-androgen contraceptives potentially reducing the risk of gestational diabetes, while DMPA injection raised concerns about elevated glucose risk. Contraceptive users also exhibited a 30% lower risk of high-risk pregnancies and births, though statistical significance was limited. Additionally, certain contraceptives were linked to adverse maternal health outcomes, such as ectopic pregnancy and pregnancy-related venous thromboembolism, emphasizing the need for careful consideration and monitoring when selecting contraceptive methods. These findings underscore the nuanced relationship between contraception and maternal health, calling for ongoing research and informed decision-making to optimize family planning strategies.

### Implications for practice

There are several general practical implications for practice when it comes to achieving these goals:

One of the key ways to prevent maternal mortality and increase modern contraceptive use is by providing access to quality reproductive health services, including family planning, antenatal care, and skilled birth attendants. These services should be affordable, accessible, and culturally appropriate to encourage women to use them.

*Improving education and awareness*: Educating women, men, and communities on the benefits of family planning and the risks associated with maternal mortality is crucial to improving reproductive health outcomes. This can include community outreach programs, media campaigns, and school-based education.

*Empowering women*: Women’s empowerment, including access to education, economic opportunities, and decision-making power, has been shown to improve reproductive health outcomes, including reducing maternal mortality and increasing contraceptive use. Strategies that promote women’s empowerment should be incorporated into efforts to improve reproductive and maternal health.

*Strengthening health systems*: Strong health systems are necessary to provide quality reproductive health services and respond to emergencies that may arise during pregnancy and childbirth. This includes training health care providers, ensuring adequate infrastructure, and having a robust referral system for emergency obstetric care.

*Implications for research*

There are several areas where future studies on maternal morbidity and modern contraceptive use could focus. These include:

1. Long-term studies: Future studies could focus on long-term outcomes of maternal morbidity and modern contraceptive use, including effects on maternal health and child outcomes.
2. Comparative studies: Comparative studies could be conducted to compare the effectiveness of different modern contraceptive methods in reducing maternal morbidity and mortality.
3. Intervention studies: Intervention studies could be conducted to evaluate the impact of specific interventions, such as increased access to family planning resources or targeted education programs for healthcare providers, on maternal morbidity and modern contraceptive use.
4. Cost-effectiveness studies: Studies could be conducted to evaluate the cost-effectiveness of different modern contraceptive methods in preventing maternal morbidity and mortality.
5. Qualitative studies: Qualitative studies could be conducted to explore the barriers to modern contraceptive use in LMICs and to identify strategies for overcoming these barriers.

Systematic reviews are an essential tool for synthesizing existing evidence on maternal health and modern contraceptive use. Here are some recommendations for future systematic reviews in this area:

- *Include studies from low- and middle-income countries*: Maternal health and contraceptive use are particularly important in low- and middle-income countries, where access to reproductive health services is often limited. Future systematic reviews should prioritize including studies from these settings, which may have different healthcare systems, cultural norms, and social determinants of health.
- *Assess the quality of evidence*: It is important to assess the quality of the evidence included in systematic reviews, to determine the strength of the conclusions drawn from the review. Future systematic reviews should use appropriate tools to assess the quality of the studies included and provide clear explanations of their judgments.
- *Consider different types of interventions*: There are many different types of interventions that can improve maternal health and modern contraceptive use, including community-based interventions, educational programs, and policy changes. Future systematic reviews should consider a range of intervention types and compare their effectiveness to identify the most promising approaches.
- Analyze subgroup effects: Maternal health and contraceptive use are influenced by many factors, including age, parity, and socioeconomic status. Future systematic reviews should explore subgroup effects, such as whether interventions are more effective for certain populations, to identify interventions that are most effective for specific subgroups.

Consider the broader health system context: Maternal health and contraceptive use are influenced by the broader health system context, including financing, governance, and the availability of health workers. Future systematic reviews should consider how health system factors may influence the effectiveness of interventions and provide recommendations for addressing these broader systemic issues.

Address equity considerations: Maternal health and contraceptive use are often shaped by gender norms and power imbalances. Future systematic reviews should consider the equity implications of interventions and provide recommendations for addressing gender inequalities and promoting women’s empowerment.

## Supporting information

S1 Appendix Data sources. (DOCX)

S2 Appendix Search strategy. (DOCX)

S3 Appendix Data extraction form. (DOCX)

S4 Appendix Characteristics of the studies. (DOCX)

S5 Appendix Acronyms. (DOCX)

S1 Fig Flow diagram. (PDF)
