# Supplementary material for: A meta-analysis into the mediatory effects of family planning utilization on complications of pregnancy in women of reproductive age
Source: PLoS One. 2024 Mar 18;19(3):e0294475. doi: 10.1371/journal.pone.0294475 (PMC10947693; doi:10.1371/journal.pone.0294475)
Supplement: S1 Appendix — (DOCX) [file pone.0294475.s001.docx]

**Appendix 1: *Data sources***

**Global databases**

Cumulative Index to Nursing and Allied Health Literature (CINAHL)

<https://www.ebsco.com/products/research-databases/cinahl-database>

OVID Medline

<https://www.wolterskluwer.com/en/solutions/ovid/ovid-medline-901>

Embase

<https://www.embase.com>

PsycINFO

<https://www.apa.org/pubs/databases/psycinfo>

Maternity & Infant Care

<https://www.midirs.org/resources/maternity-and-infant-care-mic-database/>

Clinical trial.gov

<https://clinicaltrials.gov/>

Web of science

<https://clarivate.com/webofsciencegroup/solutions/web-of-science/>

Scopus

<https://www.scopus.com>

Cochrane Central Register of Controlled Trials (CENTRAL)

<https://www.cochranelibrary.com/central>

**Local databases**

Africa (AIM)

<http://indexmedicus.afro.who.int/>

Latin America and the Caribbean (LILACS)

<http://bases.bvs.br/>

A network of Health Science Libraries across Asia (HELLIR)

<http://www.hellis.org>

Virtual Health Sciences Library

<http://www.emro.who.int/HIS/VHSL/>

IBECS

<http://ibecs.isciii.es>

Scientific Electronic Library Online (SciELO)

<http://www.scielo.br>

Pan American Health Library (PAHO)

<https://www.paho.org>

WHO Library (WHOLIS)

<http://dosei.who.int>

Western Pacific Region Index Medicus (WPRO)

<http://www.wprim.org>

Index Medicus for the South‐East Asia Region (IMSEAR)

<http://imsear.hellis.org>

Indian medical journals (IndMED)

<http://indmed.nic.in>

Native Health Research Database

<http://hscssl.unm.edu/nhd/>

**Other databases**

International Union for the Scientific Study of Population (IUSSP)

<http://iussp.org/>

Population Association of America (PAA)

<https://www.populationassociation.org>

International Conference on Family Planning (ICFP)

<http://www.fpconference2013.org/>

Department for International Development (DFID)

<https://www.gov.uk>

Canadian funding for international development projects (CIDA)

<https://www.international.gc.ca>

U.S. Agency for International Development (USAID)

<https://www.usaid.gov>

**University databases for thesis and technical reports**

The London School of Hygiene & Tropical Medicine (LSHTM) Database

<https://www.lshtm.ac.uk/>

Harvard University Database

<https://library.harvard.edu>

University of California Berkley Database

<https://www.lib.berkeley.edu>

George Washington University Database

<https://library.gwu.edu>

**Websites of relevant societies and institutions**

World Health Organization (WHO)

<https://www.who.int>

Guttmacher institute

<https://www.guttmacher.org>

Department for International Development (DFID)

<https://www.gov.uk>

U.S. Agency for International Development (USAID)

<https://www.usaid.gov>

Marie Stopes International (MSI)

<https://www.msichoices.org/>

Population Services International (PSI)

<https://www.psi.org>

United Nations Population Fund (UNFPA)

<https://www.unfpa.org>

Population council

<https://www.popcouncil.org>
