# Supplementary material for: A meta-analysis into the mediatory effects of family planning utilization on complications of pregnancy in women of reproductive age
Source: PLoS One. 2024 Mar 18;19(3):e0294475. doi: 10.1371/journal.pone.0294475 (PMC10947693; doi:10.1371/journal.pone.0294475)
Supplement: S2 Appendix — (DOCX) [file pone.0294475.s002.docx]

**APPENDICES**
***Appendix 2: Search Strategy***

Ovid MEDLINE(R) <1946 to June Week 2 2022>

After clean up : 4028 citations

1 exp Family Planning Services/ 25935

2 exp Family Planning Policy/ 1713

3 exp Contraception Behavior/ or exp Contraception/ 33264

4 exp Contraceptive Agents/ 77488

5 exp Contraceptive Devices/ 26629

6 exp Reproductive Control Agents/ 301777

7 exp Medroxyprogesterone Acetate/ 5027

8 "planned pregnanc* ".ab,ti. 630

9 "one child polic* ".ab,ti. 221

10 "pro natalist polic* ".ab,ti. 25

11 "pronatalist polic* ".ab,ti. 88

12 "antinatalist polic* ".ab,ti. 16

13 "contracept*".ab,ti. 67354

14 "fertility inhibition".ab,ti. 99

15 "fertility control".ab,ti. 1420

16 immunocontraception.ab,ti. 266

17 "birth interval* ".ab,ti. 1326

18 "birth spacing".ab,ti. 773

19 "pregnancy interval* ".ab,ti. 318

20 "condom*".ab,ti. 21419

21 "intrauterine device* ".ab,ti. 5491

22 (iud* or ius).ab,ti. 9890

23 "family plan* ".ab,ti. 21417

24 abortifacient.ab,ti. 981

25 "reproductive control* ".ab,ti. 183

26 "cervial cap* ".ab,ti. 1

27 "coiled spring* ".ab,ti. 52

28 "vaginal ring* ".ab,ti. 1026

29 "vaginal shield* ".ab,ti. 2

30 diaphragm.ab,ti. 26306

31 dmpa.ab,ti. 1196

32 "depo provera".ab,ti. 758

33 1 or 2 or 3 or 4 or 5 or 6 or 7 or 8 or 9 or 10 or 11 or 12 or 13 or 14 or 15 or 16 or 17 or 18 or 19 or 20 or 21 or 22 or 23 or 24 or 25 or 26 or 27 or 28 or 29 or 30 or 31 or 32 415489

34 exp Maternal Mortality/ or exp Maternal Death/ 11549

35 exp Reproductive Health/ 4596

36 exp Diabetes Mellitus/ 481100

37 exp Heart Diseases/ 1227628

38 exp Obesity/ 245183

39 exp Disseminated Intravascular Coagulation/ 11493

40 exp Eclampsia/ 4570

41 exp Embolism, Air/ 5864

42 exp Thromboembolism/ 61917

43 "maternal death".ab,ti. 3642

44 "maternal mortality".ab,ti. 10084

45 "maternal morbidity".ab,ti. 4942

46 "reproductive health".ab,ti. 14655

47 diabetes.ab,ti. 520825

48 "heart disease".ab,ti. 153977

49 ("sickle cell" adj2 crisis).ab,ti. 752

50 obesity.ab,ti. 243609

51 "disseminated intravascular coagulation".ab,ti. 9698

52 eclampsia.ab,ti. 15145

53 "air embolism".ab,ti. 3334

54 thromboembolism.ab,ti. 37051

55 34 or 35 or 36 or 37 or 38 or 39 or 40 or 41 or 42 or 43 or 44 or 45 or 46 or 47 or 48 or 49 or 50 or 51 or 52 or 53 or 54 2224662

56 33 and 55 30784

57 exp Cohort Studies/ 2357853

58 exp Case-Control Studies/ 1327532

59 exp Cross-Sectional Studies/ 428924

60 "cohort stud* ".ab,ti. 238169

61 "cross section* ".ab,ti. 400976

62 "case control".ab,ti. 127221

63 "observaton*".ab,ti. 20

64 57 or 58 or 59 or 60 or 61 or 62 or 63 3074896

65 56 and 64 5628

66 limit 65 to (english language and humans) 5193

67 66 not RCT.mp. [mp=title, abstract, original title, name of substance word, subject heading word, floating sub-heading word, keyword heading word, organism supplementary concept word, protocol supplementary concept word, rare disease supplementary concept word, unique identifier, synonyms] 5185

68 67 not comparative.mp. [mp=title, abstract, original title, name of substance word, subject heading word, floating sub-heading word, keyword heading word, organism supplementary concept word, protocol supplementary concept word, rare disease supplementary concept word, unique identifier, synonyms] 4502
