# Supplementary figures and images for: A meta-analysis into the mediatory effects of family planning utilization on complications of pregnancy in women of reproductive age
Source: PLoS One. 2024 Mar 18;19(3):e0294475. doi: 10.1371/journal.pone.0294475 (PMC10947693; doi:10.1371/journal.pone.0294475)

Appendix 3: Data Extraction Form


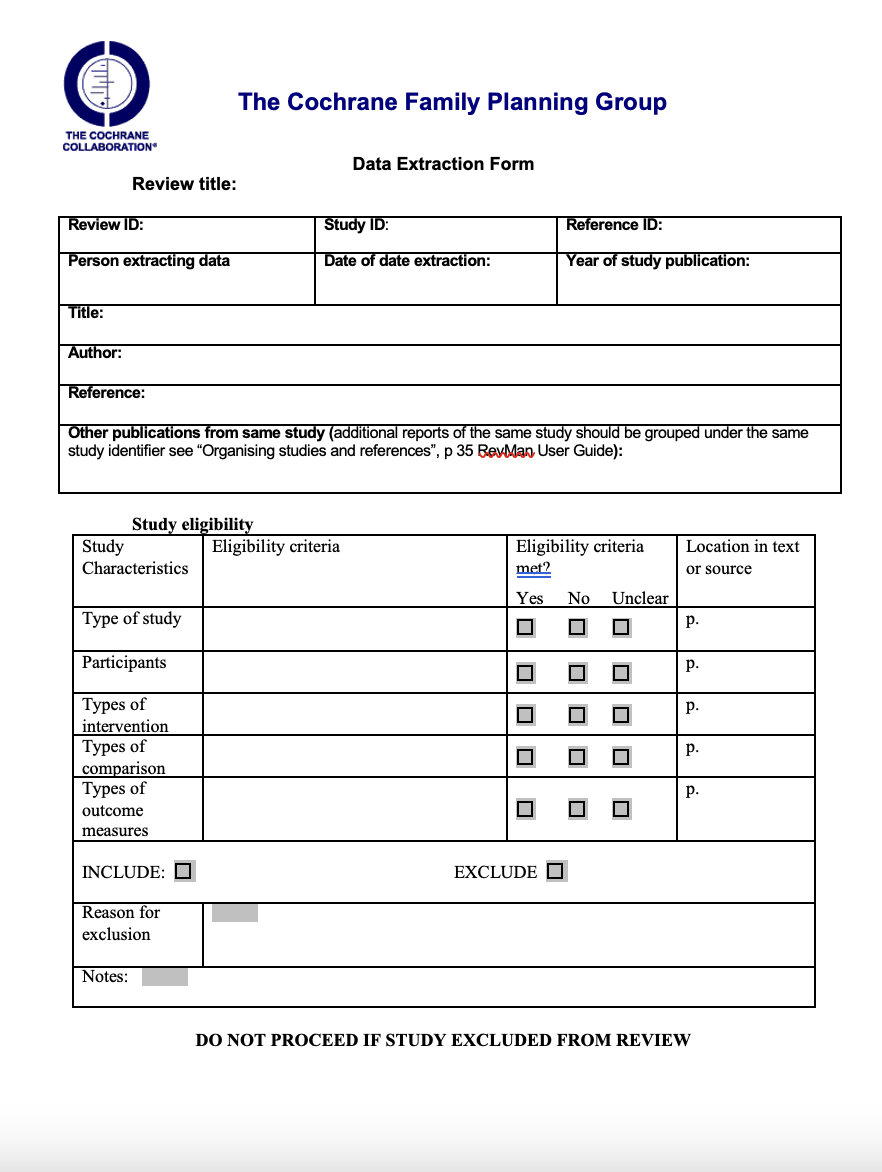


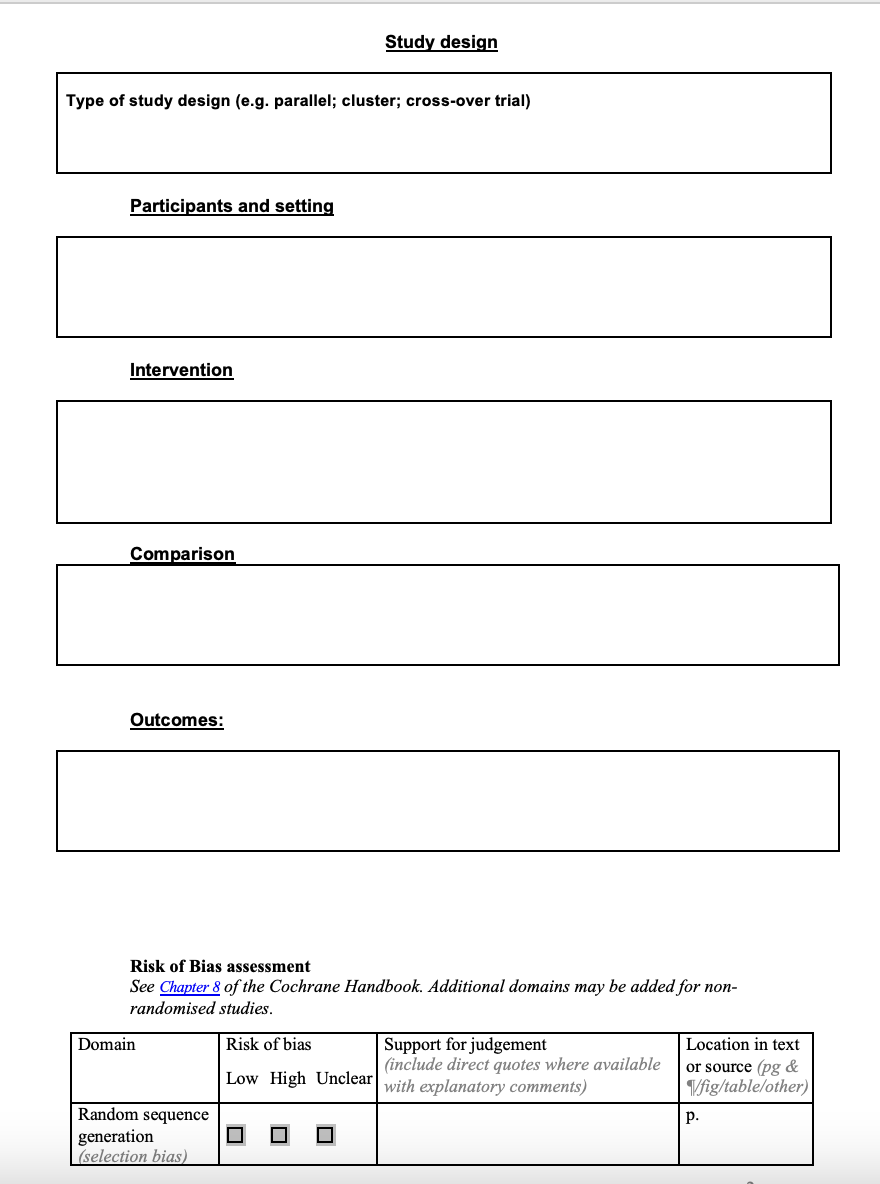


**
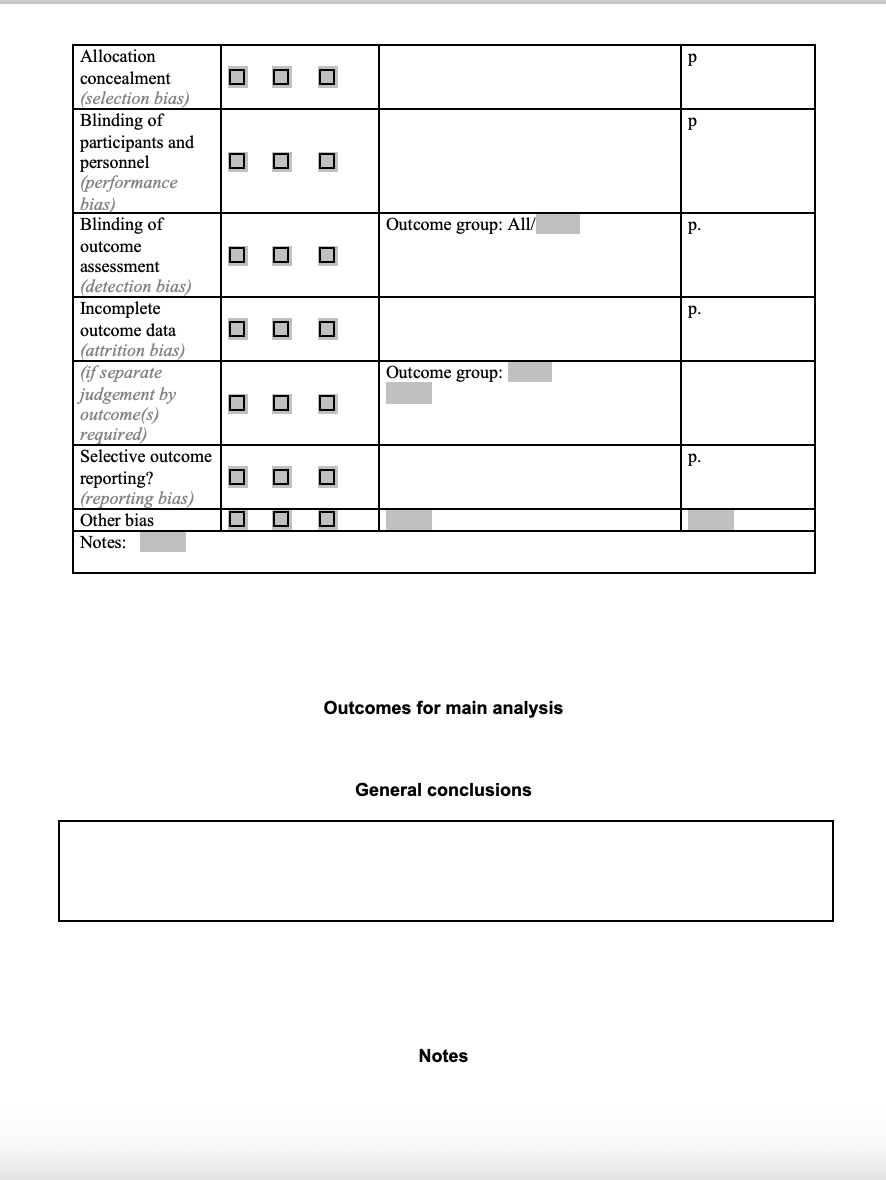

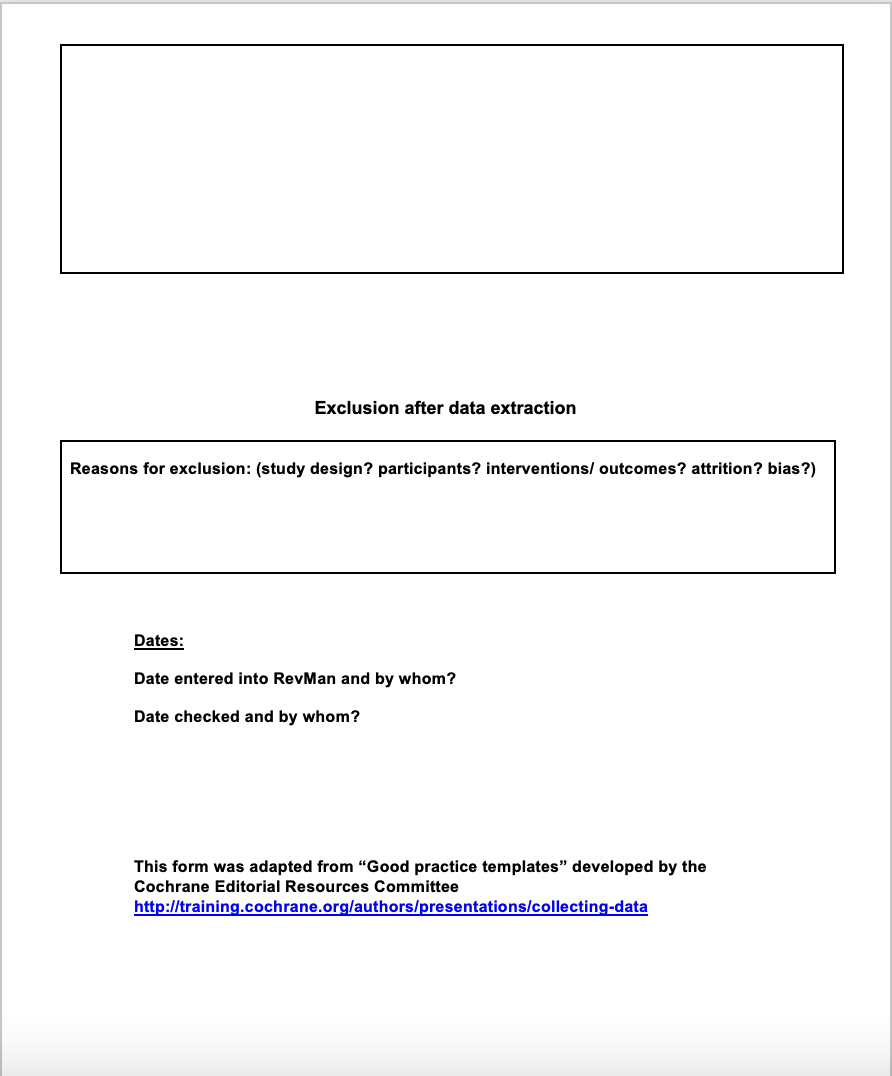
**

Supplement: S3 Appendix — (DOCX) [file pone.0294475.s003.docx]
