# Supplementary material for: A meta-analysis into the mediatory effects of family planning utilization on complications of pregnancy in women of reproductive age
Source: PLoS One. 2024 Mar 18;19(3):e0294475. doi: 10.1371/journal.pone.0294475 (PMC10947693; doi:10.1371/journal.pone.0294475)
Supplement: S4 Appendix — (DOCX) [file pone.0294475.s004.docx]

**Appendix 4: Characteristics of the studies**

| **Title** | **Country** | **Study setting (public /private or rural/urban)** | **Study design** | **Type of contraception, dosage and route of adminstration** | **Sample size** | **Study aim** | **Population** | **Outcome** | **Period of observation (weeks, months, years)** | **Findings (effect size and 95%CI)** |
| --- | --- | --- | --- | --- | --- | --- | --- | --- | --- | --- |
| Abdalhabib 2021 | Sudan | Mixed | Case-Control | Oral contraceptive use | 396 | Investigate the relationship between contraceptive use and VTE during pregnancy. | Pregnant women | Venous thromboembolism | Jan 2018-Aug 2019 | Patients with a history of contraceptive use were more susceptible to developing VTE (33.8%). |
| Bahamondes 2014 | Brazil | Mixed | Retrospective cohort | Any use of any modern contraception | 25015 | Investigate the relationship between all contraceptive use and DALY averted. | Women aged 12-50 | DALY averted | 10 years | Over the last 10 years of evaluation, provision of LARC methods and DMPA by the clinic areestimated to have contributed to DALY averted by between 37 and 60 maternal deaths, 315 –424 child mortalities, 634 –853 combined maternal morbidity and mortality and child mortality, and 1056–1412 unsafe abortions averted. |
| Bastani 2007 | Iran | Urban | Case-Control | Any use of any modern contraception | 200 | investigate that if preconception period of seminal fluid exposure affects the prevalence of preeclampsia in primigravida women. | Primigravida women | Pre-eclampsia | 1 year | The relation of use of barrier contraceptive method and preeclampsia in primigravida women was significant, so that primigravida women using the condom or withdrawal method, are in higher risk of being preeclamptic (p = 0.007). |
| Bourke 2015 | Ireland | Mixed | Cross-sectonal | Contraceptive Use | 13760 | Investigate the relationship between contraceptive use and crisis pregnancy. | Women of reproductive age | Crisis pregnancy | 2003-2010 | Those with contraceptive use at first sexual intercourse were less likely to report the experience of a crisis pregnancy OR 0.19 [0.12-0.32] |
| Chikandiwa 2018 | Kenya and Zimbabwe | Mixed | Cross-sectonal | Any use of any modern contraception | 40250 | Investigate the relationship between contraceptive use and high risk birth and under-five mortality of the resulting child. | Women of reproductive age | High-risk birth, under five mortality | One year (2011) | Contraceptive use was protective against high risk births in Zimbabwe only (aOR: 0.79, 95% CI: 0.68–0.92) and under-five mortality in both Kenya (aOR: 0.79, 95% CI: 0.70–0.89) and Zimbabwe (aOR: 0.71, 95% CI: 0.61–0.83). |
| Chowdhury 2007 | Bangladesh | Rural | Retrospective | Any use of any modern contraception | 220,000 | Examine the effect of the Matlab Maternal and Child Health and Family Planning (MCH-FP) Program on obstetric mortality rate. | All women | Maternal mortality | 30 years | Maternal mortality fell by 68% in the ICDDR,B service area and by 54% in the government service area over 30 years. |
| Godefay 2015 | Ethiopia | Rural | Case-Control | Any use of any modern contraception | 166515 | Investigate the relationship between contraceptive use and maternal mortality | Women of reproductive age | Maternal mortality | Unclear | Ever using contraceptives was independently and significantly associated with lower risks of maternal mortality. Not using contraceptives before pregnancy was associated with a higher risk for maternal mortality. OR 2.58 [1.37-4.85] |
| Gupta 2018 | Papau New Guinea | Rural | Retrospective cohort | Contraceptive impant type | 618 | Investigaate the relationship between contraceptive implant use and maternal mortality/high risk pregnancy. | Women aged 15-49 | Change in crude birthrate, change in rate of maternal and neonatal morbidity and mortality, age specific mortality rate | 2010-2016 | The introduction of the contraceptive implant on Karkar Island was associated with a fall in number of high risk pregnancy characteristics, associated with an average change in trends of 0.19 per year for severe hemorrage (95% CI 0.15-0.21) and 0.6 per year for pospartum infection (95% CI 0.40-0.71) |
| Hedderson 2007 | United States | Mixed | Case-Control | Any use of any modern contraception | 14235 | Investigate the relationship between contraceptive use and gestational DM. | Third trimester pregnant women | Gestational diabetes mellitus | Unclear | Compared with women who had not used hormonal contraceptives, women who used low androgen hormonal contraceptives for at least 6 months during the 5 years before the index pregnancy had a 16% reduced risk of GDM (covariate-adjusted odds ratio 0.84 [95% CI 0.58–1.22]). In contrast, there was a suggestion that women who used a high-androgen hormonal contraceptive for at least 6 months during the 5-year period before pregnancy had an increased risk of GDM (1.43 [0.92–2.22]). Among women who used Loestrin, the highest androgen oral contraceptive, the risk was greater (1.99 [0.91–4.32]) than for women who used other high-androgen hormonal contraceptives (1.21 [0.75–1.97]) |
| Lech 2005 | Poland | Mixed | Retrospective | Combined hormonal contraceptive pills | 363 | The aim of the study was to check and present data on the relation between combined oral contraceptives (COC) use and body weight in young women living in Poland. | Women of reproductive age | Overweight and obesity | 1 month | COC use is not associated with weight gain in young women with a Central European life-style. |
| Li 2015 | China | Urban | Case-Control | Contraceptive use (previous and current) | 4827 | Investigate the relationship between contraceptive use and ectopic pregnancy. | Women of reproductive age | Ectopic pregnancy | March 2011-April 2013 | The study revealed that the risk of EP was associated with previous use of intrauterine devices (IUDs) (AOR = 1.72, 95% CI: 1.39-2.13). Additionally, EP risk was increased following the failure of most contraceptives used in the current cycle including IUDs (AOR = 16.43, 95% CI: 10.42-25.89), oral contraceptive pills (AOR = 3.02, 95% CI: 1.16-7.86), levonorgestrel emergency contraception (AOR = 4.75, 95% CI: 3.79-5.96), and female sterilization (AOR = 4 .73, 95% CI: 1.04-21.52) |
| Nelson 2008 | United States | Urban | Retrospective | Any use of any modern hormonal contraception | 592 | Retrospective chart review of 592 indigent, primarily Latina women who had been diagnosed with gestational diabetes, monitored for up to 24 months' postpartum. | Postpartum women | Glucose tolerance | 24 months |  |
| No author listed 1998 | Africa, Asia, Europe, Latin America | Mixed | Case-Control | Oral and injectable progestogen-only contraceptivesCombined injectable contraceptives | 3697 | Evaluate the risks of cardiovascular disease (CVD) associated with the use of oral and injectable progestogen-only and combined injectable contraceptives. | Women of reproductive age | VTE, stroke, AMI | Unclear | Overall, the adjusted OR for all CVD combined compared with nonusers of any type of steroid hormone contraceptive (SHC) associated with current use of oral and injectable progestogen-only contraceptives and combined injectable contraceptives, respectively, were 1.14 (95% CI: 0.79-1.63), 1.02 (0.68-1.54), and 0.95 (0.49-1.86). No significant changes in OR were apparent for strokes, VTE, or AMI in association with any of these types of contraception. However, a small, nonsignificant increase in OR for VTE was apparent in association with oral and injectable progestogen-only contraceptives. |
| Parker 2016 | United Kingdom | Mixed | Prospective comparative | IUD | 2960 | Investigate the relationship between IUD use and pre-eclampsia | Mothers who gave birth with 15 months of recorded medical history prior to the delivery date | Pre-eclampsia | 1993-2010 | The use of an IUD prior to pregnancy was associated with a small reduction in the risk of pre-eclampsia. OR 0.76 [0.58-0.98] |
| Petersen 2014 | Denmark | Mixed | Retrospective cohort | Combined oral contraceptive (COC) use | 985,569 person-years | Investigate the relationship between contraceptive use and VTE within the first year after pregnancy. | Women aged 15-49 | Venous thromboembolism during first year following pregnancy | 1995-2009 | In conclusion, the use of combined hormonal contraceptives after early terminated pregnancies was not detrimental, but during the puerperal period, they should be used with caution. After early terminated pregnancies, the use of combined hormonal contraceptives conferred an increased risk after seven weeks (RR: 2.3, 95% CI: 1.0-5.4) (▶ Table 2). Following childbirth, the use of combined hormonal contraceptives was associated with increased risk after 14 weeks (RR: 2.8, 95% CI: 1.5-5.3) |
| Ronsmans 1997 | Bangladesh | Rural | Retrospective | Any use of any modern contraception | 200,000 | Examine the effect of the Matlab Maternal and Child Health and Family Planning (MCH-FP) Program on obstetric mortality rate. | All women | Maternal mortality | 1976-1993 | Direct obstetric mortality declined by 3% per year (rate ratio 0·97 per year [95% CI 0·95–0.99]). Direct obstetric mortality halved between 1976–86 and 1987–89 in the northern MCH-FP area, where the maternity-care programme was initiated in 1987 (0·50 [0·22–0·99]), but showed no change in the southern MCH-FP area, which had no such intervention at that time (1·07 [0·64–1·72]). After 1990, when the programme was expanded throughout the MCH-FP area, the southern part showed a downward (non-significant)trend in direct obstetric mortality (0·68 [0·35–1·32]).However, direct obstetric mortality also declined between 1987 and 1989 in the southern comparison area (0·48 [0·26–0·83]) in the absence of an intense maternity-care programme, and remained stable thereafter. In the northern comparison area, there was no such decline in direct obstetric mortality (0·78 [0·40–1·40]). |
| Skouby 1982 | Denmark | Urban | Case-Control | Oral contraceptive use | 18 | Evaluate the carbohydrate metabolic status in 10 women with previous non-insulin-dependent diabetes in pregnancy and in 8 control subjects | All women | Glucose tolerance | 6 months | There is no impact on glucose tolerance, body weight, or blood pressure in non-insulin diabetic women or controls during post-partum hormone intake as judged by the fasting glucose and plasma glucose during an oral glucose tolerance test. |
| Shurie 2018 | Kenya | Urban | Case-Control | Use of levonorgestrel only emergency contraceptive | 316 | Investigate the relationship between levonorgestrel-only emergency contraceptive use and ectopic pregnancy. | Women of reproductive age | Ectopic pregnancy | Unclear | The use of LNG-EC pills adjusting for history of using depo Provera and menarche was associated with 9.4 times increased odds of developing ectopic pregnancy {OR: 9.34 (95% CI: 3.9 -16.0)} |
| Thadhani 1999 | United States | Mixed | Prospective cohort | Oral contraceptive use | 4308 | Investigate the relationship between oral contraceptive use and maternal morbidities hypertension, preeclampsia, and gestational hypertension. | Women reporting their first singleton pregnancy lasting > 9 mo between 1991 and 1995 | Gestational hypertension, preeclampsia | 1991-1995 | There is a an inverse relationshipin recent contraceptive use and development of gestational hypertension. There is not one with preeclampsia. Women may be at increased risk for developing preeclapmsia if they had used contraceptives within 2 years of their pregnancy or those who had used them for >8 years. OC use before pregnancy was associated with a RR for gestational hypertension of 0.7 as compared to a RR of 1 in non-users or past users [0.4-1.1]. OC use before pregnancy (recent) was associated with a RR for preeclampsia of 1.4 [0.8-2.4]. |
