# Supplementary material for: A meta-analysis into the mediatory effects of family planning utilization on complications of pregnancy in women of reproductive age
Source: PLoS One. 2024 Mar 18;19(3):e0294475. doi: 10.1371/journal.pone.0294475 (PMC10947693; doi:10.1371/journal.pone.0294475)
Supplement: S5 Appendix — (DOCX) [file pone.0294475.s005.docx]

**Appendix 5: *Acronyms***

| AUB | Abnormal uterine bleeding |
| --- | --- |
| CI | Confidence interval |
| COC | Combined oral contraceptive |
| E2 | Estradiol |
| EE | Ethinyl estradiol |
| GRADE | Grades of Recommendation, Assessment, Development, and Evaluation |
| HC | Hormonal contraceptive |
| IUD | Intrauterine device |
| LNG-IUS | Levonorgestrel intrauterine system |
| MD | Mean differences |
| OC | Oral contraceptive |
| OCP | Oral contraceptive pill |
| OR | Odds ratios |
| RR | Risk ratios |
| SMD | Standardized mean differences |
| WHO | World Health Organization |
