# Supplementary material for: A meta-analysis into the mediatory effects of family planning utilization on complications of pregnancy in women of reproductive age
Source: PLoS One. 2024 Mar 18;19(3):e0294475. doi: 10.1371/journal.pone.0294475 (PMC10947693; doi:10.1371/journal.pone.0294475)

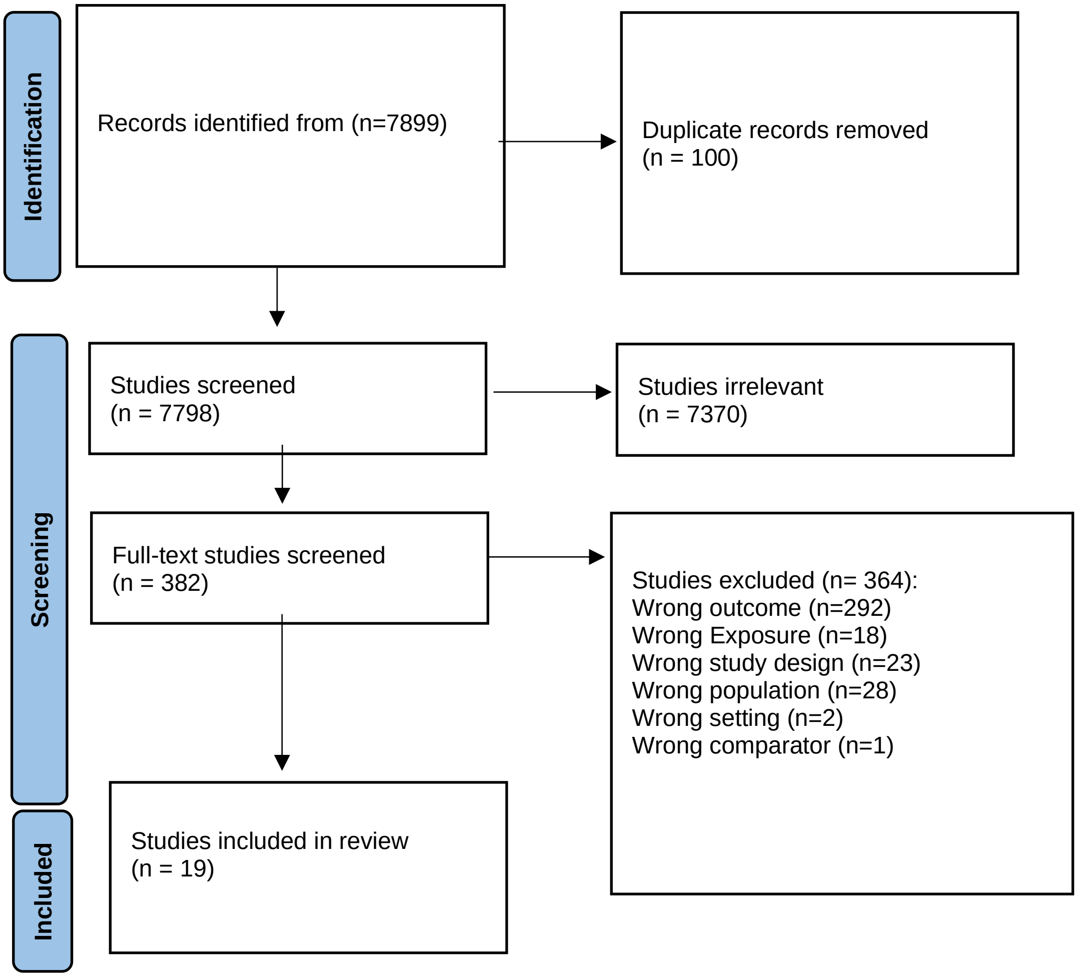

Figure 1. Flow diagram

From: Page MJ, McKenzie JE, Bossuyt PM, Boutron I, Hoffmann TC, Mulrow CD, et al. The PRISMA 2020 statement: an updated guideline for reporting systematic reviews. BMJ 2021;372:n71. doi: 10.1136/bmj.n71. For more information, visit: http://www.prisma-statement.org/

Figure 2: Pre-Eclampsia

Study or Subgroup	log[Risk Ratio]	SE	Weight	Risk Ratio IV, Fixed, 95% CI
Bastani 2007	0.4932	0.1573	27.3%	1.64 [1.20, 2.23]
Parker 2016	-0.3155	0.1029	63.8%	0.73 [0.60, 0.89]
Thadhani 1999	0.2872	0.2751	8.9%	1.33 [0.78, 2.29]
**Total (95% CI)**			**100.0%**	**0.96 [0.82, 1.13]**

Heterogeneity: Chi² = 20.07, df = 2 (P < 0.0001); I² = 90%
Test for overall effect: Z = 0.50 (P = 0.62)

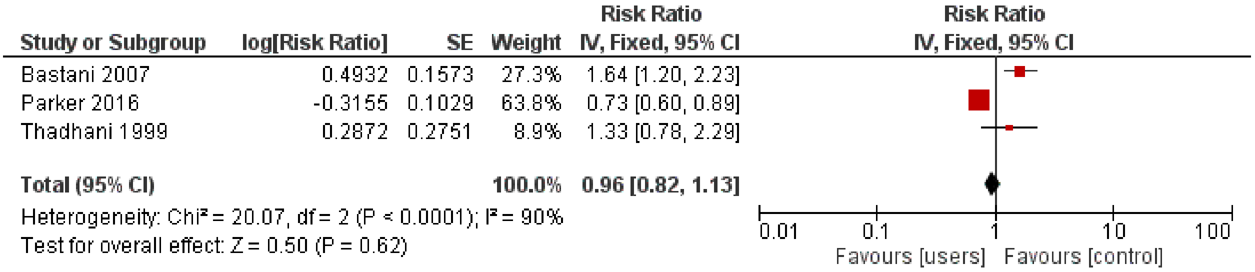

Figure 3: High-Risk Pregnancy and Birth

Study or Subgroup	log[]	SE	Weight	IV, Fixed, 95% CI
Bourke 2015	−1.6607	0.2345	9.4%	0.19 [0.12, 0.30]
Chikandiwa 2018	−0.2231	0.0755	90.6%	0.80 [0.69, 0.93]
Total (95% CI)			100.0%	0.70 [0.61, 0.80]

Heterogeneity: Chi² = 34.05, df = 1 (P < 0.00001); I² = 97%
Test for overall effect: Z = 4.98 (P < 0.00001)

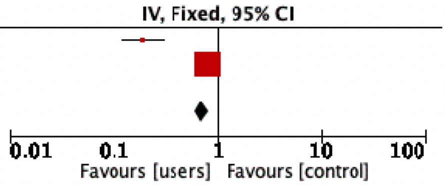

Figure 4: **Pregnancy-Related Venous Thromboembolism**

Study or Subgroup	Users Events	Users Total	Control Events	Control Total	Weight	Odds Ratio M-H, Fixed, 95% CI
Abdalhabib 2021	67	198	37	198	31.9%	2.23 [1.40, 3.54]
Anonymous 1998	70	171	635	2288	68.1%	1.80 [1.31, 2.48]
Total (95% CI)		369		2486	100.0%	1.94 [1.49, 2.52]
Total events	137		672			

Heterogeneity: Chi² = 0.54, df = 1 (P = 0.46); I² = 0%
Test for overall effect: Z = 4.96 (P < 0.00001)

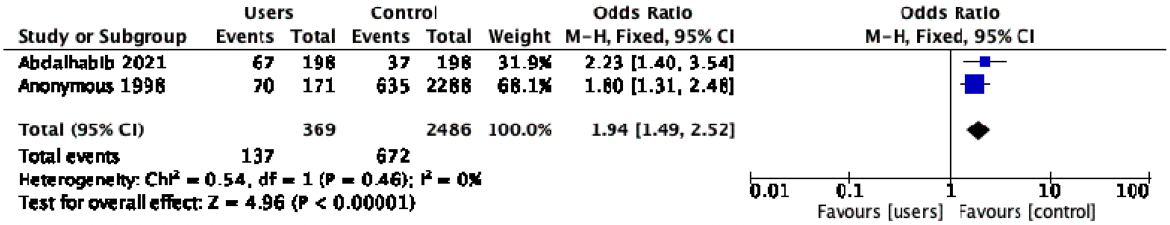

Figure 5: **Ectopic Pregnancy**

Study or Subgroup	Users Events	Users Total	Control Events	Control Total	Weight	Odds Ratio M-H, Fixed, 95% CI
Li 2015	1112	2196	1283	2594	99.0%	1.05 [0.94, 1.17]
Shurie 2018	26	79	17	237	1.0%	6.35 [3.21, 12.54]
Total (95% CI)		2275		2831	100.0%	1.10 [0.98, 1.23]
Total events	1138		1300			

Heterogeneity: Chi² = 26.15, df = 1 (P < 0.00001); I² = 96%
Test for overall effect: Z = 1.67 (P = 0.10)

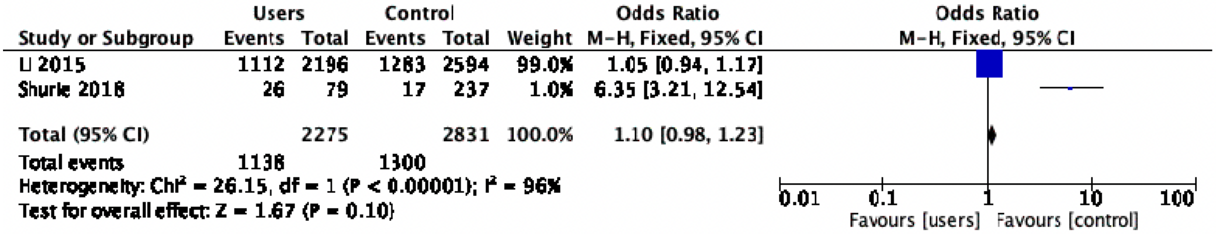

Supplement: S1 Fig — (PDF) [file pone.0294475.s006.pdf]
